# Influence of breast reconstruction on technical aspects of echocardiographic image acquisition compared with physician-assessed image quality

**DOI:** 10.1186/s40959-019-0052-7

**Published:** 2019-11-04

**Authors:** Joaquin Duarte Ow, Mohamad Hemu, Anel Yakupovich, Parva Bhatt, Hannah Gaddam, Nicole Prabhu, Ibtihaj Fughhi, Melody Cobleigh, Melissa Tracy, Louis Fogg, Tochukwu Okwuosa

**Affiliations:** 1 Department of Internal Medicine, John H. Stroger, Jr. Hospital of Cook County, Chicago, IL USA; 2 Department of Internal Medicine, Rush University Medical Center, Chicago, IL USA; 3 University of Illinois at Urbana-Champaign, Champaign, IL USA; 4 Department of Hematology/Oncology, Rush University Medical Center, Chicago, IL USA; 5 Division of Cardiology, Rush University Medical Center, 1717 West Congress Parkway, Kellogg Bldg, Suite 328, Chicago, IL 60612 USA; 6 Department of Community, Systems and Mental Health Nursing, College of Nursing, Rush University Medical Center, Chicago, IL USA

**Keywords:** Echocardiogram, Image quality, Breast reconstruction, Tissue expander

## Abstract

**Introduction:**

Assessment of cardiac function after treatment for breast cancer relies on interval evaluation of ventricular function through echocardiography. Women who undergo mastectomy more frequently choose to undergo breast reconstruction with implant. This could impede assessment of cardiac function in those with left-sided implant. We aimed to examine whether left-sided breast reconstruction with tissue expanders (TE) affect echo image acquisition and quality, possibly affecting clinical decision-making.

**Methods:**

A retrospective case-control study was conducted in 190 female breast cancer patients who had undergone breast reconstruction with TE at an urban academic center. Echocardiographic technical assessment and image quality were respectively classified as excellent/good or adequate/technically difficult by technicians; and excellent/good or adequate/poor by 2 board-certified cardiologist readers. Likelihood ratio was used to test multivariate associations between image quality and left-sided TE.

**Results:**

We identified 32 women (81.3% white; mean age 48 years) with left-sided/bilateral TE, and 158 right-sided/no TE (76.6% white, mean age 57 years). In multivariable analyses, we found a statistically significant difference in technician-assessed difficulty in image acquisition between cases and controls (*p* = 0.01); but no differences in physician-assessed image quality between cases and controls (*p* = 0.09, Pearson’s r = 0.467).

**Conclusions:**

Left-sided breast TE appears to affect the technical difficulty of echo image acquisition, but not physician-assessed echo image quality. This likely means that echo technicians absorb most of the impediments associated with imaging patients with breast TE such that the presence of TE has no bearing on downstream clinical decision-making associated with echo image quality.

## Introduction

Cardiovascular disease is an important cause of morbidity and mortality in cancer patients. In particular, chemotherapy and chest radiation therapy increase the risk of cardiotoxicity; including cardiomyopathy and heart failure, coronary artery disease, pericardial disease, valvular dysfunction, and conduction abnormalities [1–3]; and may persist up to 45 years after therapy [1].

Although advances in breast cancer treatment have reduced its toxicity and increased survival, breast cancer survivors are still at increased risk of myocardial and coronary dysfunction due to prior cancer therapy [2, 3]. When breast cancer is detected at an early stage, the treatment usually involves surgery; with options for breast preservation or mastectomy. Increasingly, women undergo mastectomy [4], either because of personal preference, hereditary predisposition to breast cancer or because magnetic resonance imaging detects multicentric disease. Women who chose mastectomy are increasingly undergoing reconstruction [4]; the most common reconstructive procedure is tissue expansion, followed by insertion of a permanent implant [5]. There is extensive research that relies on echocardiography (echo) and other cardiac imaging modalities to determine early markers of cardiotoxicity in cancer survivors [6, 7]. Echocardiography is deemed the technique of choice when undertaking a global comprehensive assessment of cardiac structure and function at baseline and during the cancer process [8]. Different guidelines have different intervals for cardiac assessment via echo in cancer survivors that can range from 6 to 18 months after the last chemotherapy [8–10].

The acquisition of good quality echocardiographic images in post-mastectomy, breast cancers survivors after left breast implant insertion is important because echocardiography is one of the most readily available and utilized tests to assess myocardial function. MUGA is another imaging modality employed in the assessment of left ventricular function in cancer patients; however, this modality increases radiation exposure as compared to echocardiographic imaging. Furthermore, unlike MUGA, echocardiography is highly comprehensive and assesses cardiac structure and function without associated radiation risk. However, the importance of echocardiography in the detection of myocardial toxicity in cancer survivors combined with the increase in breast reconstruction [11] leads us to a potential new challenge in cardiac surveillance of these patients - the interference of breast implants in the acquisition of a good acoustic window in ultrasound [12, 13].

We aimed to assess whether the effects of breast reconstruction on echocardiographic image quality could ultimately influence clinical decision-making. In this study, we examined the effects of left-sided breast reconstruction with tissue expanders (TE) on echocardiographic image acquisition; and its downstream effects on image quality.

## Methods

### Study population

We conducted a case-control study in a population of female patients, age > 18 years, with biopsy-proven breast cancer, diagnosed between 1990 and 2015, and treated surgically at Rush University Medical Center (RUMC). Surgical treatments included mastectomy/lumpectomy and reconstruction with TE. All patients had echocardiographic follow up after mastectomy. Patients with metastatic disease at diagnosis were excluded from our study. The study was approved by RUMC institutional Review Board.

### Patient selection

**Fig. 1:**
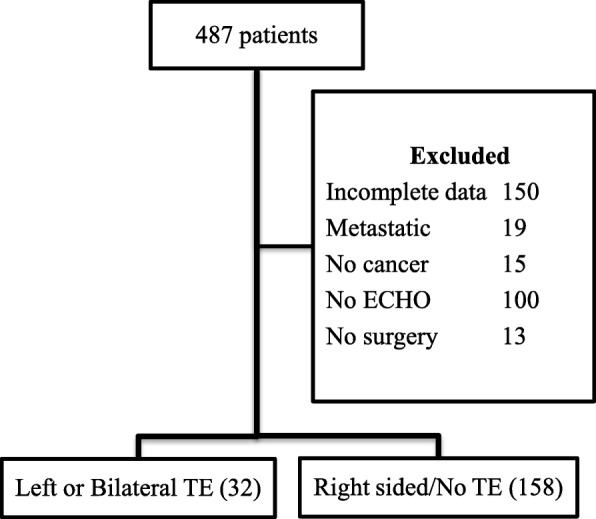
Patient selection

A total of 190 patients meeting both inclusion and exclusion criteria were identified from the electronic medical record system (EPIC) - Fig. 1. The case group consisted of patients with a left-sided TE (left-sided only or bilateral); and the control group were those without a left-sided TE (right-sided or no reconstruction).

The study variables (Table 1) were extracted from the RUMC electronic medical record and stored in the REDCap database software. After chart review was completed, the information was exported to SPSS software for statistical analysis.

**Table 1:** Baseline characteristics

	Left/Bilateral TE		Right/No recontstruction TE		*p* value*
Race					0.738
Non-white	(6)	18.7%	(37)	23.4%	
White	(26)	81.3%	(121)	76.6%	
Age at dx	48 ± 12.4		57.5 ± 11.1		**0.000**
Breast Cancer					
Primary side					
Right	(9)	28.2%	(74)	46.8%	
Left	(23)	71.8%	(79)	50%	
Bilateral	(1)	2%	(5)	3.2%	
Size					**0.010**
2–5 cm	(8)	25%	(40)	25.3%	
> 5 cm	(5)	15.6%	(6)	3.7%	
Chest or skin invasion	(1)	3.2%	(1)	0.6%	
Not specified	(1)	3.2%	(9)	5.6%	
Node status					0.329
N0	(14)	43.7%	(83)	52.5%	
N1	(18)	56.3%	(73)	46.2%	
Grade					0.633
I	(3)	9.4%	(22)	14.2%	
II	(18)	25%	(41)	26.5%	
III	(16)	50%	(56)	36.1%	
Not specified	(7)	15.6%	(36)	23.2%	
TE side					
Left	(14)	7.4%			
Right			(7)	3.4%	
Bilateral	(18)	9.4%			
No reconstruction			(151)	79.4%	
Treatment					
Anthracyclines	(28)	90.2%	(104)	70.7%	**0.049**
Antiestrogen	(25)	78.1%	(100)	68%	0.248
Trastuzumab	(29)	90.6%	(105)	70.9%	**0.012**
Radiation Therapy	(29)	90.6%	(104)	71.2%	**0.001**
ECHO					
LVEF	58 ± 5		58 ± 7		0.913
BMI	26.9 ± 5.4		28.7 ± 7.3		0.242
SBP	123 ± 18		131 ± 22		0.051
DBP	71 ± 10		73 ± 12		0.350
Comorbidities					
COPD	–		(8)	5%	**0.016**
Prior Chest surgery	(3)	9.7%	(13)	8.4%	0.826
HTN	(16)	51.6%	(87)	56.5%	0.618
DM	(3)	9.4%	(25)	16.2%	0.300
Dyslipidemia	(8)	25%	(49)	32%	0.427
Heart failure	(2)	6.5%	(17)	11%	0.419
CKD	(1)	3.2%	(4)	2.6%	0.852
CAD	(1)	3.2%	(17)	11%	0.278
Obesity	(17)	32.7%	(73)	36.9%	0.879
Medications					
ASA	(7)	21.9%	(45)	29.2%	0.390
ACE/ARB	(8)	25%	(38)	24.7%	0.969
Beta blocker	(8)	25%	(47)	30.5%	0.528
Statin	(9)	28.1%	(44)	28.8%	0.942
Smoking					0.615
Never smoker	(16)	53.3%	(80)	57.6%	
Former Smoker	(11)	36.7%	(52)	37.4%	
Active Smoker	(3)	10%	(7)	5.9%	

*p values < 0.05 are listed in boldface

### Tissue expanders characteristics

TE used were constructed from silicone elastomer made of polydimethylsiloxane. These expanders were inflated with saline to obtain the desired size prior to implantation. TE were available in either smoothed or textured surface shells. They were made of cohesive gel that holds uniformly together and takes the natural shape of the breast (model Mentor MemoryShape). The majority of patients had TE implanted in a retro-pectoral position.

### Echocardiographic data

Echocardiographic images were obtained using four major positions: the apical, parasternal, suprasternal notch and subcostal views. This was based on the standard set of views as recommended by the American Society of Echocardiography [14]. Studies were performed by highly skilled echocardiography technicians with long standing experience at a tertiary care center. All examinations were conducted using GE (Vivid-7) and Phillips (IE-33) echocardiographic machines.

### Echocardiographic image quality assessment

Data regarding the technician assessment of the technical difficulty of the echocardiographic image acquisition was obtained from the report; excellent, good, adequate, technically difficult (Table 2). The echocardiographic images were subsequently independently reviewed and rated by 2 board certified cardiologists who were blinded to the patient’s history and technician rating. The image qualities were assigned by the cardiologists as a rating of excellent, good, fair, or poor image quality (Table 2). The scales used by both the technicians and cardiologists are subjective.

**Table 2:** Technician and cardiologists assessment scale^a^

Technical difficulty (Technicians)	Image quality (Cardiologists)
Excellent	Excellent
Good	Good
Adequate	Fair
Technically difficult	Poor

^a^Technicians assess technical difficulty of image acquisition, cardiologists assess image quality

## Statistical analysis

Baseline characteristics were obtained for each of the study groups. Categorical variables were summarized as percentages and absolute numbers; and continuous variables as mean ± standard deviation. Echocardiographic technical assessment and image quality were respectively classified as excellent/good or adequate/technically difficult by technicians; and excellent/good or adequate/poor by 2 board-certified cardiologist readers. Interpretations were derived from sonographer assessed technical difficulty of the study, and 2 board-certified cardiologists’ assessment of image quality. Likelihood ratio was used to test associations between image quality and left-sided TE. The results were adjusted for age, anthracycline/trastuzumab use, radiotherapy, COPD and size of tumor; since these covariates were shown to be different between study groups. Agreement between cardiologists was determined by Pearson’s correlation coefficient. The data was streamlined to report findings from one of the cardiologist echo readers. Statistical significance was set at *p* < 0.05.

## Results

There were 32 cases and 158 controls. Table 1 shows the baseline characteristics of the study groups. The patient population was mostly white (77%). The mean age was 48 ± 12 for the study group and 57 ± 11 for the control group; and the mean BMI for the study group was 26.9 ± 5.4, and 28.7 ± 7.3 for the control group. There was no difference between groups in tumor grade, node status, smoking status, LVEF, medications and most comorbid conditions.

There were significant differences in age, tumor size, COPD status and treatment received; specifically, anthracyclines, radiotherapy and trastuzumab between groups.

**Fig. 2:**
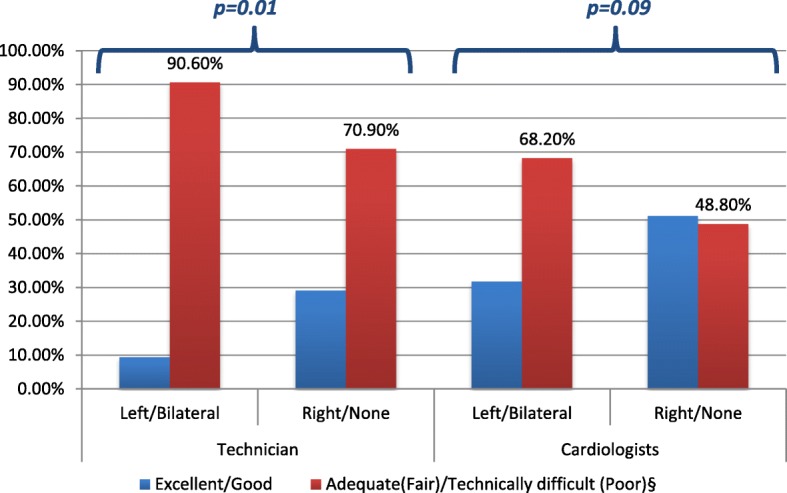
Image Quality As Assessed by Technician or Cardiologist in Patients with Tissue Expanders*.*Adjusted for age, tumor size, COPD status and cancer treatment received. § Parentheses represents image quality by cardiologists

The technicians rated 9.4% cases as excellent/good and 90.6% as adequate/technically difficult; and rate 29.1% controls as excellent/good and 70.9% as adequate/technically difficult (LR = 6.47, *p* = 0.01) – Fig. 2/Table 3. On the other hand, the cardiologists rated 31.8% cases as excellent/good and 68.2% as fair/poor; and rated 51.2% controls as excellent/good and 48.8% as fair/poor (LR = 2.88 *p* = 0.09) – Fig. 2/Table 3. Multivariate analysis showed a relationship dependent on implant side (left/bilateral vs right/none) even when adjusted for age, tumor size, COPD status and treatment received (anthracyclines, radiotherapy and trastuzumab). The Pearson’s correlation coefficient (r) between both cardiologist readers = 0.467. Our study findings were essentially unchanged when image quality data from the second echo reader were used for analysis.

**Table 3:** Image quality ratings^a^

	Technician		Cardiologist	
	Excellent/Good	Adequate/ technically difficult	Excellent/Good	Fair/Poor
Left/Bilateral	9.4%	90.6%	31.8%	68.2%
Right/None	29.1%	70.9%	51.2%	48.8%

Adjusted for age, tumor size, COPD status and treatment received (anthracyclines, radiotherapy and trastuzumab

^a^Expressed as percentage of cases or controls

## Discussion

We found significant differences in the technical difficulty of image acquisition as rated by echo technicians when comparing left/bilateral TE to a right-sided/no TE group. Interestingly, this did not translate to physician-assessed image qualities of the same studies. As such, we found no significant difference in image quality as assessed by cardiologists when comparing left/bilateral TE to a right-sided/no TE group.

Our study is unique in that we evaluated both the technical difficulty of the acquired studies as assessed by the echo technicians, as well as image quality reviewed by our experienced cardiologist echo readers. To our knowledge, this is the first study to have examined both in patients post breast reconstruction; and possibly has implications for clinical decision-making. In addition, our study focused on breast tissue expanders particularly (rather than all reconstructive techniques).

Our results are different from the findings from a previous study by Pignati et al. [15] in which patients with left-sided/bilateral tissue expanders had significantly poorer image quality. In that retrospective study, from 2000 to 2012, 44 women post breast reconstruction after mastectomy for breast cancer, were divided in 2 groups: left/bilateral vs right breast implant (control group). In the study group, it was judged as adequate in only 50% of cases (15 patients) vs. 100% of the controls (*p* < 0.001). There could be a number of explanations for the differences in both study findings. Ours was a larger study including more patients. Furthermore, our study was more rigorous and parsimonious as the echocardiographic image qualities in our study were assessed by only 2 independent cardiologists.

As mentioned, many women are choosing breast reconstruction after mastectomy as evidenced by an increase in breast reconstruction procedures [5]. This trend raised an important clinical question in the surveillance of this patient population since the effects of breast implants on the quality of acquired echo images have been described in case reports [12, 13]. We decided to study this effect by assessing not only the impact of TE on the acquisition of good-quality echocardiographic images, but also the effects on physician interpretation.

The heart is located in the left pectoral area; as such, most of echocardiographic imaging involves the left chest. It therefore follows that procedures that involve the left-side of the chest (including left breast, whether purely left-sided or bilateral) are more likely to impact cardiac imaging, while no chest procedures or those that purely involve the right breast or right side of the chest are not.

Our study suggests that while the technicians found the presence of breast TE to increase the technical difficulty for performance of the studies, they were able to adequately complete the studies such that the cardiologist echo readers were able to visualize the cardiac structures and rate them as better image quality. This may suggest that well-trained sonographers can overcome the intricacies of echocardiographic imaging associated with breast TE, and thereby prevent possible inadequate and incompetent clinical decision-making as a consequence of poor quality images. This has particular relevance in the population of patients with breast cancer whom might receive cardiotoxic chemotherapy such as anthracyclines and/or trastuzumab as well as radiation therapy, in whom (sometimes frequent) cardiac monitoring with echocardiography is paramount.

In our study, the case group had larger tumors and younger ages and accordingly received more anthracyclines, radiotherapy and trastuzumab. Larger and aggressive tumors are more likely to undergo systemic therapy, and less likely to undergo surgery. Multivariate analysis showed a relationship dependent on implant side (left/bilateral vs right/none) even when adjusted for age, tumor size, COPD status and treatment received (anthracyclines, radiotherapy and trastuzumab). A separate analysis was run with the ungrouped categories of technical assessment and image quality (Table 3); no significant difference was found between technical assessment or image quality and study group.

The limitations of our study include the number of patients excluded due to lack of information during the initial screening, lack of randomization, and the retrospective nature of the study. Another limitation is the fact that the scales utilized for technical difficulty and image quality assessment are based on subjective assessments. The categories were grouped to simplify reporting and increase the amount of subjects per group.

## Conclusion

Left-sided breast TE appears to affect the technical difficulty of echocardiographic image acquisition, but not physician-assessed echocardiographic image quality. This likely means that echo technicians absorb most of the imaging complexities associated with breast TE, resulting in far less significant implications of breast tissue expanders on downstream clinical decision-making associated with echocardiographic image quality. This likely has significant implications for quality of care in breast cancer patients who often require echocardiographic monitoring of cardiovascular structure and function due to prior chemo- and/or radiation therapy.

## Data Availability

Yes
